# Evaluation of Nanoparticle Penetration in the Tumor Spheroid Using Two-Photon Microscopy

**DOI:** 10.3390/biomedicines9010010

**Published:** 2020-12-24

**Authors:** Feby Wijaya Pratiwi, Chien-Chung Peng, Si-Han Wu, Chiung Wen Kuo, Chung-Yuan Mou, Yi-Chung Tung, Peilin Chen

**Affiliations:** 1 Research Center for Applied Sciences, Academia Sinica, Taipei 11529, Taiwan; febywijaya@gate.sinica.edu.tw (F.W.P.); vp@gate.sinica.edu.tw (C.-C.P.); kuo55@gate.sinica.edu.tw (C.W.K.); tungy@gate.sinica.edu.tw (Y.-C.T.); 2 Graduate Institute of Nanomedicine and Medical Engineering, Taipei Medical University, Taipei 11031, Taiwan; smilehanwu@tmu.edu.tw; 3 Department of Chemistry, National Taiwan University, Taipei 10617, Taiwan; cymou@ntu.edu.tw

**Keywords:** mesoporous silica nanoparticles, spheroid, penetration, physicochemical properties, two-photon microscopy

## Abstract

Mesoporous silica nanoparticles (MSNs) have emerged as a prominent nanomedicine platform, especially for tumor-related nanocarrier systems. However, there is increasing concern about the ability of nanoparticles (NPs) to penetrate solid tumors, resulting in compromised antitumor efficacy. Because the physicochemical properties of NPs play a significant role in their penetration and accumulation in solid tumors, it is essential to systematically study their relationship in a model system. Here, we report a multihierarchical assessment of the accumulation and penetration of fluorescence-labeled MSNs with nine different physicochemical properties in tumor spheroids using two-photon microscopy. Our results indicated that individual physicochemical parameters separately could not define the MSNs’ ability to accumulate in a deeper tumor region; their features are entangled. We observed that the MSNs’ stability determined their success in reaching the hypoxia region. Moreover, the change in the MSNs’ penetration behavior postprotein crowning was associated with both the original properties of NPs and proteins on their surfaces.

## 1. Introduction

Tumor-targeting nanoparticle (NP) drug delivery systems have been proven to be promising in advanced anticancer treatment because the anticancer drugs can be delivered to the tumor sites specifically, reducing the side effects and toxicity to the healthy cells [1]. Despite these exciting results for NPs as anticancer drug nanocarriers, their efficacy is still limited; only a few of them meet clinical expectations [2]. The primary reason for such compromised antitumor efficacy is the restricted penetration and accumulation of NPs in solid tumors, which prevents nanomedicines penetration into tumors after extravasation from the blood vessels. Consequently, many NPs that show promising results in vitro are not effective in the in vivo environment. Therefore, to optimize the delivery efficiency of NPs in vivo, it is important to systematically study the influence of the physicochemical properties of NPs for penetration and accumulation in environments with pathological tumor characteristics (namely, dense extracellular matrix (ECM) and high interstitial fluid pressure).

In the past decades, many research efforts have focused on the design of NP delivery systems capable of penetrating deep into tumors [3,4,5,6]. One of the most promising NPs in the tumor-targeting delivery system is the mesoporous silica nanoparticle (MSN), which possesses many desirable properties for nanocarriers such as tunability of size and surface functionality, controllable property, biocompatibility and facile surface modification [7,8,9,10,11,12]. It has been generally considered that the properties of NPs (size, surface charge, shape, chemical composition, crystal structure, elasticity, physicochemical stability, surface area, surface energy and surface roughness) play a critical role in their cellular uptake and penetration into the tumor [13,14,15,16,17,18,19]. Though the interrelationship between these properties has been extensively investigated, no reliable predictions have been reached. Most studies are based on univariate evaluation and, thus, fail to present the tiered perspectives of their relationship. Additionally, the crosstalk between individual physicochemical properties has received the least attention [13,15]. Therefore, establishing a systematic evaluation of the correlations between the essential NPs’ physicochemical properties and their transport in the tumor would be particularly informative for guiding the design of nanocarrier systems.

Before proceeding with real in vivo experiments, it is desirable to test MSNs with various physicochemical properties in a simplified model system mimicking the in vivo microenvironments of tumors [20]. The tumor spheroid is an emerging model system that mimics tumors with collapsed lymphatic drainage and contains a high-level ECM, making it similar to NP penetration barriers in the in vivo environments. The presence of spatial cell-cell interactions and the gradient distribution of oxygen, nutrients and metabolites in the spheroid convincingly reflect multidrug resistance [20,21,22]. Additionally, spheroids provide several advantages such as cell clonality, facile maintenance, and feasibility of long-term monitoring, making them a potential platform for nanomedicine testing [23,24].

In this study, the role of MSNs’ sizes and surface charges in their penetration and accumulation within tumor spheroids was examined using two-photon imaging, which allows for noninvasive and deeper quantitative imaging of NP events in real-time throughout the spheroid matrix, with low phototoxicity and sufficient resolution [11,25]. To interpret the heterogeneity of results, a hierarchical clustering analysis (HCA) was employed by integrating MSN properties, transport effects and their correlation coefficients [11,26,27]. The established library helped decipher the model transportation of various MSNs and estimated the critical factors limiting their efficacy. It was, therefore, useful in selecting suitable MSNs to carry specific drugs to target tumor regions.

## 2. Experimental Methods

### 2.1. Synthesis of MSNs with Different Physicochemical Properties

Different sizes of polyethylene glycol (PEG) MSNs containing rhodamine B isothiocyanate (RITC), or fluorescein isothiocyanate (FITC) (25, 50, and 200 nm) were synthesized using a Stöber-like process developed by Mou et al. [7,8,28]. FITC or RITC-conjugated 3-aminopropyltrimethoxysilane (APTMS) was freshly prepared by stirring 5 mg of FITC or 8 mg RITC in 99.5% ethanol (5 mL) contained APTMS in the dark overnight. Then, 4.2 mL FITC or 2.5 mL RITC-APTMS was incorporated alongside tetraethyl orthosilicate (TEOS) in the silica matrix during MSN formation. The size of MSNs could be precisely regulated by controlling the total ammonia concentration at a specific temperature. Different surface modifications, including PEG, N-Trimethoxysilylpropyl-N, N, N-trimethylammonium chloride (TA), and 3-trihydroxysilyl propyl methyl phosphonate (THPMP) were incorporated into MSNs to form neutral, positively, and negatively charged MSNs, respectively. PEG silane and a mixture of PEG-silane (1.12 mmol) and TA-silane (0.54 mmol) were added consecutively to the colloidal solution while stirring to produce FITC or RITC-labeled MSN/PEG and MSN-PEG/TA particles, respectively. For the hydrothermal treatment, the as-synthesis solution (~50 mL) was kept in the oven at 70 °C and 90 °C for the first and second days, respectively. Afterward, the surfactant templates in the pores of the MSNs were extracted using hydrochloric acid (37%). The resulting products were then washed thrice in ethanol, collected by centrifugation and stored in 99.5% ethanol. To obtain dyes-labeled MSN-PEG/THPMP, the MSN-PEG was further modified with THPMP-silane (2.2 mmol). The size, hydrodynamic diameter and zeta-potential were measured for each type of NP. Unless otherwise stated, all the chemicals were obtained from Sigma-Aldrich (St. Louis, MO, USA), Across Organics (Waltham, MA, USA), or Fisher Scientific (Rochester, NY, USA).

### 2.2. The Characterization of Physicochemical Properties of MSNs

The size of the MSNs was determined using transmission electron microscopy (TEM) (Hitachi H-7 at 100 kV, Tokyo, Japan). For sample preparation, the dispersed MSNs (≈1.5 µg/mL in ethanol) were dropped on carbon-coated Cu grids (400 mesh) and dried in air. The obtained micrographs were analyzed with the Fiji ImageJ software (National Institutes of Health, Bethesda, MD, USA). Dynamic light scattering and the ζ-potential of the MSN were measured using a Zetasizer Nano ZS (Malvern instruments, Malvern, UK).

The fluorescence stability of MSNs was measured under a slightly acid pH medium (pH ~6.5–6.7) to evaluate their physicochemical stability under the influence of the tumor’s microenvironment. 25 µg/mL of MSNs were dispersed in a slightly acid culture medium. Measurements were taken directly after the MSNs were mixed with the medium and after 16 h incubation under an excitation wavelength of 530 nm using a Spectra Max M21102 UV–vis spectrophotometer (Molecular Devices, San Jose, CA, USA). The percentage change of the fluorescence signal at 590 nm represented the stability change of the MSNs. In addition, the hydrodynamic size of the MSNs under the same conditions (at 0 and 16 h) was measured to evaluate the size and dispersity change.

### 2.3. Generation of MG-63 Spheroids

Before the introduction of cells, 96-well round-bottom microplates (Nunc 268200, Thermo Fisher Scientific Inc., Rochester, NY, USA) were treated with 5% (*v*/*v*) Synperonic^®^ F-108 (07579, Sigma Aldrich, Co., St Louis, MO, USA) and kept overnight to create low attachment well plates for the cells. Prior to cell seeding, the F-108 solution was removed from each well and the round-bottom wells were washed with sterilized water three times. Then, the well plates were air-dried for 20 min in the hood.

For the 3D spheroid generation, MG-63 (60279, Bioresource Collection and Research Center, Hsinchu, Taiwan) cells were seeded into the F-108-treated ultra-low attachment U-shaped wells at a density of 3125 cells per well in a volume of 100 µL. The 96-well plates were placed in the humidified cell incubator at 37 °C, with 95% air and 5% CO_2_ atmosphere. After 1-week of incubation, the MG-63 spheroids were processed with nanoparticle experiments.

### 2.4. Evaluation of the Penetration of MSNs in a Spheroid

To study the penetration of MSNs in spheroids in the absence of serum, the spheroid in 96-well plates was treated under a serum starvation medium for 30 min at 37 °C. After serum starvation, RITC-labeled MSNs in a free serum medium at 500 μg/mL concentration in a final 100 μL volume were incubated with the spheroid for 16 h at 37 °C. Before imaging, spheroids were washed thrice with PBS to stop the uptake of MSNs and remove unbound MSNs, and then fixed in 4% paraformaldehyde (PFA). The same procedure was used to evaluate NP penetration in a serum-containing medium. In addition, to evaluate the contribution of passive diffusion in NP penetration, the spheroids were fixed using PFA 4% overnight before incubation with MSNs.

To obtain the spatial distribution of MSNs in the spheroids, z-stack images were captured from the surface up to the center of the spheroids (i.e., at a depth of ≈160 μm) using a two-photon microscope (FVMPE-RS, Olympus, Tokyo, Japan) at an excitation wavelength of 900 nm with a water-immersion objective (Plan N W MP 25 × 1.05 NA 2 mm WD, Olympus). For the quantitative analysis of NP penetration in the spheroids, the sectioned images were processed using MATLAB R2014b (The MathWorks, Inc., Natick, MA, USA) and Fiji ImageJ software (National Institutes of Health, Bethesda, MD, USA). A radial distribution script was used to calculate the average pixel intensity at various distances from the center of the spheroid. The average pixel intensity at each distance was subsequently multiplied by 2 πr to determine the total pixel intensity at each distance. The integrals of these total pixel intensities are equal to the respective area’s total pixel intensity. Moreover, to quantify the distribution of MSNs in a different spheroid region, we calculated the total pixel intensity in four concentric volumes (in which the volume of each region was the same). Region 1 was the volume of the spheroid’s innermost, followed by regions 2 and 3, while region 4 was the spheroid’s volume in the outer layer. The detailed calculation of this region is presented in Supporting information.

### 2.5. Statistical Analysis

The results are represented as the mean ± standard deviation (SD) obtained from multiple samples (N = 4–6) with at least two or more experiments. For univariate statistical analysis, one-way ANOVA analysis was used to determine the statistical differences among the experimental groups (cutoff *p*-value < 0.05). Multivariate statistical analysis was used to determine the relationships in the entire raw data. The correlation analysis was performed on the normalized value of each experimental group based on the Spearman coefficient (ρ). It was considered that variables were strongly correlated when ρ ≥ 0.7 or ρ ≤ −0.7 and moderately correlated at 0.6 < ρ < 0.7 or −0.7 < ρ < −0.6 (cutoff *p*-value < 0.05) [29]. Moreover, hierarchical cluster analysis was performed based on the Euclidean distance (Ed) of the observable groups and/or between different NPs based on physicochemical properties. The furthest neighbor algorithm was used to link each column or row into clusters.

## 3. Result

### 3.1. Effect of MSNs’ Properties on the Total Penetration and Accumulation in the Spheroids

In this study, free scaffold MG-63 cell monoculture was employed to investigate NP penetration in a 3D model. Even though no exogenous matrix was added in the cell culture, it was reported that the cells could secrete their own ECM when the spheroid forms [21,30,31]. To demonstrate that our spheroids contained ECM, we stained the spheroids with ECM-related proteins such as fibronectin, collagen IV and vitronectin (Figure S1). From the images, the ECM molecules were found to surround the spheroids, which was very similar to the microenvironments around solid tumors.

To evaluate the effect of the physicochemical properties of NPs on their transport into the spheroids, we investigated a set of nine different MSNs (25, 50, and 200 nm MSN with PEG, TA, and THPMP), thus allowing for a comparative study. The transmission electron micrograph (TEM) image, hydrodynamic size, surface charges and other physicochemical properties are shown in the supporting information Figure S2 and listed in Table S1. Before proceeding with the NP penetration study in the spheroid, MSN uptake in the monolayer (2D) cell culture was examined. Figure S3 shows that all MSNs could be efficiently taken up by the cells (>70%) regardless of surface functionalization.

**Figure 1:**
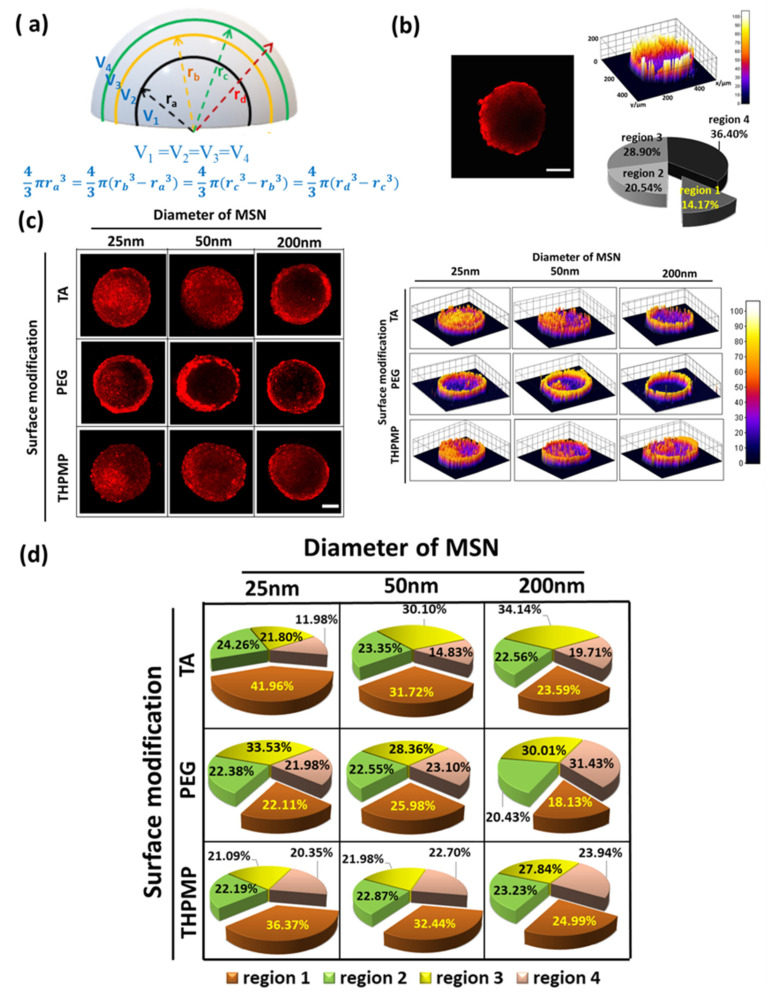
Penetration and distribution of free dye and mesoporous silica nanoparticles (MSNs) in spheroids. (**a**) Illustration of different concentric regions in the spheroid. (**b**) Two-photon image of rhodamine B isothiocyanate (RITC) in spheroid (z = 160 µm) and the distribution of RITC. Scale bars = 100 μm. N = 4. (**c**) Two-photon images of different MSNs in spheroids (z = 160 µm) and the distribution of MSNs. Scale bars = 100 μm. (**d**) Accumulation of MSNs in the different concentric regions. N = 4.

The penetration and accumulation of RITC-labeled MSNs in the spheroid were monitored by a two-photon microscope. Integrated fluorescence intensity was normalized before comparison to account for minor variations in the spheroids’ sizes and allowed for the direct comparison of the total association of NPs within the spheroids. To quantify the distribution of MSNs in a different spheroid region, the total pixel intensity in four concentric volumes (in which the volume of each region is the same) were calculated. Figure 1a illustrates the locations of different regions in the spheroid. Region 1 is the volume of the spheroid’s innermost (r_a_), followed by rim volume in region 2 (between r_a_ and r_b_) and 3 (between r_c_ and r_b_), while region 4 is the rim volume in the outer layer. Region 1, which showed the maximum fluorescence intensity variations of hypoxia dye (related to the quiescent and necrotic cell region), was designated as the core [32].

As a control experiment, we first studied the penetration of free RITC dye. After 16 h of incubation, free RITC showed high accumulation in the peripheral region (Figure 1b) but rarely appeared in the center region of the spheroid (region 1). Though small molecules can easily penetrate within the matrix, they are prone to be eliminated from the tumor. In contrast, negatively and positively charged MSNs with diameters of 25 and 50 nm were distributed evenly in all spheroid regions, as shown in Figure 1c. Indeed, these NPs could deliver a higher amount of RITC into the core of the spheroids (>30%) (Figure 1d) compared to the free dyes (14%).

Upon examination of various MSN images at different incubation times, our results indicated that only the negatively and positively charged MSNs with diameters of 25 and 50 nm could accumulate in region one after 16 h. On the other hand, MSNs with a diameter of 200 nm showed high intensity only on the outer layers, which indicated that these MSNs were taken up by the cells but were restricted from penetrating deeper cell layers even after 24 h of incubation (Figure S4). Though both smaller MSN-TA and MSN-THPMP could penetrate deeper than the larger ones, their accumulation was also influenced by the NPs’ surface charges (MSN-TA > MSN-THPMP) [33]. On the other hand, the fluorescence of MSN-PEG, regardless of the size, was rarely observed in the core region (region 1). Moreover, the percentage of individual cells of the spheroid that took up MSN-TA, MSN-PEG and MSN-THPMP were measured to be 70.93, 44.30 and 67.68%, respectively (Figure S5).

Notably, the pH gradient in the tumor microenvironment must be considered in the design of the NPs. We investigated the role of MSN physicochemical stability by measuring the fluorescence intensity change in a slightly acid medium (pH~6.5–6.7), which mimicked the tumor microenvironment’s pH. The change in fluorescence stability was related to the MSN’s chemical and/or physical changes, such as the homogeneity, degradation and aggregation of NP in the solution [34]. The degree of NP dispersity change under a slightly acid medium is shown as the polydispersity index (PDI) values presented in Table S2. The fluorescence change of all MSNs was plotted toward their accumulation in the spheroid’s core (Figure S6). These data revealed a correlation between NP stability and accumulation in the deeper region. This result explained that MSN-PEG, irrespective of size, remained in the spheroid periphery due, in part, to poor stability in the deeper tumor environment where the larger polydisperse index (PDI) was observed for MSN-PEG in the acidic environment, as shown in Table S2.

### 3.2. Effect of MSNs’ Properties on Diffusive Motion in the Spheroids

To improve our understanding of the influence of NP physicochemistry in passive diffusion to their accumulation in spheroids, we further adopted the zombie cell method developed by Warren et al. [35,36]. Spheroids were fixed overnight under 4% PFA to ensure that all the cellular dynamics and cellular uptake processes were halted, allowing only NP diffusion to dominate the penetration process.

**Figure 2:**
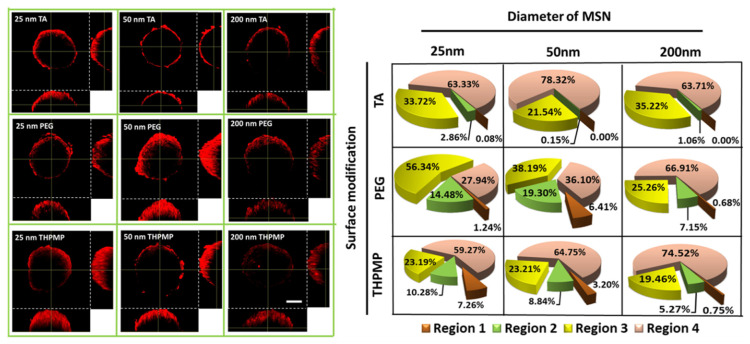
Two-photon images and distribution of MSNs in the middle section of fixed spheroids. Scale bar = 100 µm. N = 4.

Figure 2 depicts that only MSN-THPMP and MSN-PEG with diameters of 25 and 50 nm were detected in the deeper regions, whereas all MSNs with a diameter of 200 nm remained in the outer layer of the spheroids. However, the positively charged MSN-TA, regardless of size, preferred to reside on the outer layer of the spheroid, indicating that not only the size but also the charges of the NP surfaces contribute significantly to passive diffusion.

### 3.3. Accumulation of MSNs in the Hypoxic Region of the Spheroid

Once the spheroid’s diameter increased beyond 200 µm, a hollow-core sphere structure was formed containing proliferating cells in an exterior layer, and quiescent cells with limited oxygen and nutrition supply in the necrotic core, which resulted in a hypoxic region [37]. In this experiment, oxygen-sensitive dye (image-iT Red) was employed to map the oxygen variation in the spheroid. The red fluorescence from this dye started to be detected when oxygen concentration was less than 5%. To examine NPs and hypoxia region colocalization, green color MSN containing FITC with similar properties to RITC MSN was synthesized. Because the function of FITC or RITC dye was only to visualize MSN behavior, their penetration behavior would not be affected by replacing the dyes provided since they have similar properties of size and charge.

**Figure 3:**
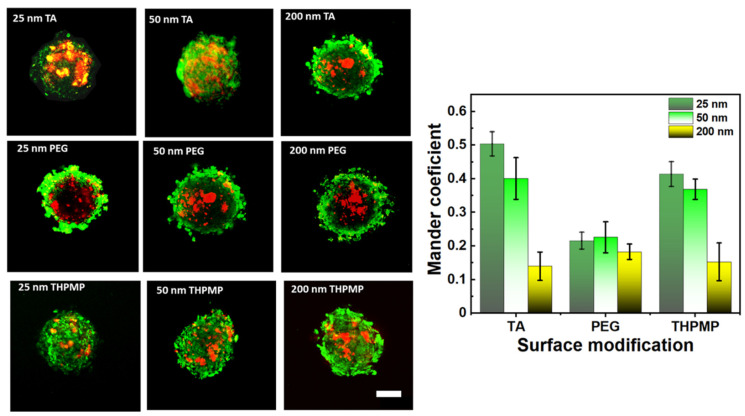
Two-photon images and distribution of MSNs (green) in the middle section of spheroids stained with Image-iT Red oxygen-sensitive reagent (red). Scale bar = 100 µm. N = 4.

Figure 3 shows the accumulation preference of MSNs in the hypoxia region of the spheroid. The degree of MSN (green) overlapping with the hypoxia region (red) was quantified based on Mander’s overlapping coefficients. The Mander’s coefficients measure the amount or degree of colocalizing pixels or voxels in each color channel; in other words, it accounts for the numbers of MSN FITC that overlap with image-iT red dyes. The increased colocalization of green fluorescence MSNs (FITC-labeled MSN) with image-iT red dyes indicated their capability to reach and reside in the spheroid’s hypoxic region (Figure 3) [32,38]. MSN-TA and MSN-THPMP (25 nm and 50 nm) showed a higher overlapping index with the hypoxic region than other types of MSNs, which corresponded to their ability to overcome the physiological barriers leading to deep penetration and uptake into the tumor’s hypoxic and necrotic areas.

### 3.4. Profiling the Multihierarchical Physicochemical Properties of MSNs in Their Transport

Unlike the standard method, which investigates only a specific physicochemical property, we investigated the influence of size, surface charge and stability simultaneously on the penetration of MSNs in this experiment. Here, size, surface charge and the absolute number of the surface charge (|surface charge|) were defined from the hydrodynamic size and zeta potential value of MSNs (Table S1), respectively, while the stability was obtained by measuring the MSN’s fluorescence change after 16 h of incubation in a slightly acid culture medium (Figure S6). The imaging features related to penetration behavior introduced in the previous sections (Figure 1, Figure 2 and Figure 3), and the NPs’ intracellular fates described in the supporting information (Figure S7), were used to determine five observable changes in the heat map. For example, the NPs’ accumulation percentage in the spheroid’s core region under normal conditions (Figure 1), the NPs’ accumulation percentage in the core region of the spheroid under fixation (Figure 2) and the NPs’ colocalization percentage with the hypoxia region (Figure 3) defined the core accumulation, passive diffusion and hypoxia preference values, respectively. Moreover, the intracellular fate related to penetration, including lysosome entrapment and endosomal recycling values, was described from the colocalization percentage of NPs with the lysosome and the Rab 11 markers, respectively (Figure S7). To avoid having too broad range values, we used a standard procedure to normalize each element’s value (z) so that the average for each group after normalization was 0, and the standard deviation was 1 (Table S3) [39].

**Figure 4:**
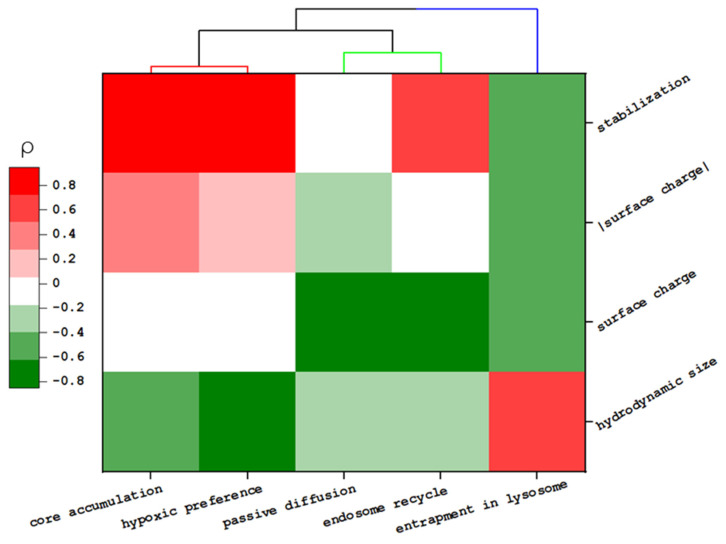
Heat map of the relationship between the physicochemical properties of MSNs and their transportation behavior. The color map represents the Spearmen coefficient value (ρ).

Furthermore, the correlation between physicochemical properties in the transportation behaviors and accumulation in spheroids were calculated based on Spearman’s correlation coefficient (ρ) (Table S4). The dendrogram heat map of these values was established to visualize and evaluate these multiparameter data (Figure 4 and Figure S9a). Moreover, HCA was applied to search for similarities of NPs in the feature space or transportation events by measuring their Euclidean distance (Ed) [40,41].

Though we noted that specific surface modification enhances the total accumulation of NP in the core region (i.e., MSN-TA and MSN-THPMP > MSN-PEG), there was no direct correlation between the surface charge value and the penetration depth of the spheroid (ρ_surface charge_ < 0.1 or ρ_|surface charge|_ < 0.4). Instead, this property influenced some specific transportations, such as passive diffusion (ρ = −0.61) and early endocytic recycling (ρ = −0.85) [33,42,43]. Moreover, partly, penetration of NPs was inversely correlated with the NP size (ρ = −0.62), which is in good agreement with previous reports [44,45]. Among MSNs’ properties, the NPs’ stability played a more dominant role in the total accumulation of NP in the core (ρ = 0.95 with *p* < 0.01).

HCA confirmed the close relationship between NP accumulation in the tumor’s core and the hypoxic region (Ed = 1.08). Thus, these MSNs, which easily reached the tumor’s core, could potentially be used to transport hypoxia-activated prodrugs in solid tumors. Moreover, this analysis pointed out the similarity between NPs. Our results indicated that for small NPs, the similarity in their penetration behavior would be close to each other when NPs have a similar surface charge (25 nm MSN-TA similar to 50 nm MSN-TA or 25 nm MSN-THPMP similar to 50 nm MSN-THPMP). However, such a relationship is not applicable to larger NPs.

### 3.5. The Influence of Serum on MSN Penetration in the Spheroid

**Figure 5:**
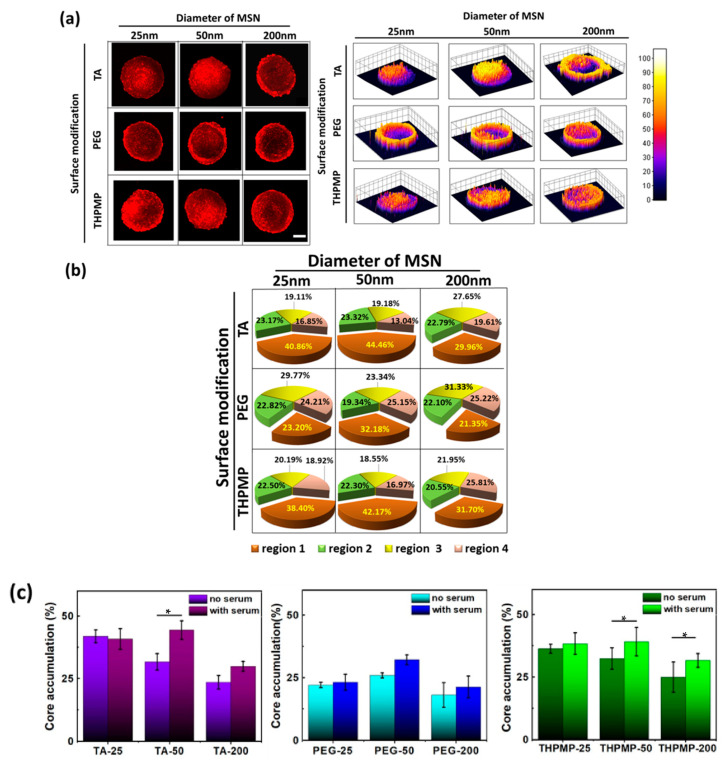
Penetration and distribution of MSNs in spheroids in the presence of serum. (**a**) Two-photon images of MSNs in spheroids and their distribution (z = 160 µm). Scale bars = 100 μm. (**b**) Accumulation of MSNs in different spheroid’s regions. N = 4. (**c**) comparison of the accumulation percentage of MSNs in the spheroid’s core with or without serum (* *p*-value < 0.05).

The effect of serum on NP penetration in the spheroid was also investigated in this experiment [46]. The images and distributions of various MSNs in the spheroid in the serum-containing medium are depicted in Figure 5. The enhanced accumulation of MSNs in the core region was only observed for 50 and 200 nm, while 25 nm MSNs showed no substantial changes in their properties after 16 h of incubation in media with serum and 50 nm and 200 nm MSNs exhibited an increase in hydrodynamic size and changes in the surface charges, suggesting that the corona formation contributes to the overall size of NPs (Figure S8a). The difference in zeta potential indicated that the protein corona also influenced NP surface charges (Figure S8b). While the original properties of MSN define the corona protein formation, the HCA in Figures S9b and S10 confirmed that the penetration enhancements of NPs post-protein coronation were associated with the NPs’ pristine and post-protein coronation properties.

## 4. Discussion

In the monolayer cell culture, most NPs, regardless of surface functionalization, were efficiently taken up by cells (>70%), which, due to all the monolayer cells, could directly interact with the NPs without the abundant ECM and cell barrier (Figure S3). However, in the 3D model, the outer cell layers and the ECM network hindered the outside particle transport and formed a more realistic mass transfer gradient. 

Due to the restrained convection in the tumor’s microenvironment, the NPs’ penetration in solid tumors was driven mainly by diffusion. Therefore, smaller MSNs moved faster toward the core of the solid tumor, whereas larger ones (200 nm) were too large to move through the tissue matrix (average mesh size < 100 nm). This obtained result is partly in good agreement with a previous report [15,16]. Though size significantly regulates passive diffusion, the retention of particles in tumor tissues is another critical factor in the accumulation of NPs in tumors [15], which explains why free RITC dye molecules did not accumulate more in the core region of the tumor spheroid than MSNs with diameters of 25 nm and 50 nm.

For multilayer cellular structures, endo-exocytosis or transcytosis could be another essential factor in the accumulation of NPs in the core region of the solid tumors [3]. For instance, though 200 nm MSNs could be effectively endocytosed by cells in the outer layer of the spheroid [47], many of them ended up in the lysosome, with fewer chances for transcellular transport to reach the deeper regions of the tumor (Figure S7). Indeed, the balance between the NPs’ cell interaction and their migration is the vital point that defines the penetration process [42,48]. NP surface charge influences both the binding and penetration resistance arising from the charge field within the spheroid (cell membrane and ECM protein). The repulsion between negatively charged NPs (MSN-THPMP) and cell membranes decreased their probability of cellular uptake. However, at the same time, this force pushed them inward into a deeper region through the matrix space, leading to better accumulation in a deeper part of the spheroid [48]. In contrast, the firmer interaction of MSN-TA with the negatively charged cell membrane and other ECM components could translate into low diffusion via an extracellular mechanism. However, the intracellular fates of MSN-TA that facilitated the transcellular transport favored deeper penetration [4,8,49].

Regardless of the size and surface modification of NPs, their stability in the tumor microenvironment is the most important factor governing the accumulation of NPs in the core of solid tumors. Although all the MSNs used in this experiment exhibited high stability neutral culture medium or saline solution, a slight decline in environmental pH could alter the original properties of MSN-PEG, leading to a decrease in the homogeneity (dispersion) in the solution and compromising their stability during transport. On the other hand, the MSN-THPMP and MSN-TA (25 and 50 nm) possess higher positive or negative charges, which creates electrostatic repulsion between individual particles. Therefore, they exhibited better physical stability even at a slightly acid pH solution [50,51,52].

Moreover, the stabilization of NP is not solely affected by surface charge but also shape, size, capping agents, and other physicochemical properties [53]. Indeed, our and other’s previous reports showed that smaller particles were more stable over a wider pH range because of more robust interactions between the nanoparticles and the surface coating or capping agent [52,54]. Overall, the balance of MSNs and cell interactions, the transcellular process, passive diffusion between the cells and the ECM matrix and the MSNs’ stability during the transport process determines their penetration abilities.

Moreover, once NPs are exposed to the serum medium, the adsorption of the biomolecules present in the environment decreases their high bare surface free energy, resulting in the formation of the so-called protein corona, which significantly reforms the original NPs’ identity [48,55]. However, the formation of the protein corona on small NPs is not so significant due to their interparticle porosity and curvature, where the kinetics of evolution of proteins is faster, resulting in rapid equilibrium and a thinner (incomplete) protein layer as judged by the lack of significant change in the NPs’ hydrodynamic size in the media with serum [56]. While MSN’s original properties determine protein corona formation, the NPs’ penetration enhancement is associated with the NPs’ properties postprotein coronation [57]. Therefore, although MSNs were not conjugated with any specific ligand, some serum component protein (such as transferrin, albumin), which took over the MSNs’ surface, might reduce the destination of MSNs to the lysosome, leading to an increase in the exocytosis and transcytosis process for deeper penetration [4]. Because different physicochemical MSNs attract various proteins, it is necessary to study the corona construction on each type of MSN and investigate which component significantly influences the transportation and penetration of MSNs in the spheroid.

## 5. Conclusions

We investigated the physicochemical properties and penetration relationship profile of MSNs using HCA in a spheroid model. This profile allowed for the easy visualization of the contributions of the MSNs’ three basic properties concerning their diverse transportation involved in NP accumulation in a deeper tumor region. Simultaneously, it allowed us to analyze the NPs’ behavior similarities, thereby making it possible to identify the categories of NPs. Our results suggested that the size and surface charge uniquely influence the transport of different NPs. Therefore, they should be studied together to accurately evaluate the penetration of nanocarriers in the tumor microenvironment. We also observed that the critical factor for MSNs to reach the tumor core and hypoxic region was closely related to their stability. Additionally, both the NPs’ original physicochemistry and corona proteins determined their capability for penetration postserum coronation. Our results help us understand the NPs’ penetration behavior in tumor spheroids, which can ultimately be utilized in selecting suitable MSNs to carry drugs to target specific tumor regions. Finally, the HCA method can be extended beyond NPs’ properties and penetration to other NP biointeractions and nanotherapeutic effects in tumors and other disease models.

## Data Availability

The data presented in this study are available on request from the corresponding author.

## Supplementary Materials

The following are available online at https://www.mdpi.com/2227-9059/9/1/10/s1, Figure S1: The immunostaining of cell spheroid with various protein marker commonly found in ECM, Figure S2: TEM micrograph of MSNs, Figure S3: The confocal images (a) and flowcytometry (b) of different MSN uptake in monolayer cell culture, Figure S4: Time-dependent MSN’s penetration into the core region of the spheroid, Figure S5: The confocal image of individual cells obtained from spheroids, Figure S6. Stabilization of MSNs in slightly acid (a) free medium and (b) serum-containing medium at 0 (black line) and 16 h (red line), Figure S7: Colocalization of MSNs with (a) lysosome marker and (b) Rab11 (recycling endosome marker), Figure S8: The MSN’s (a) hydrodynamic size and (b) zeta potential change in the serum-containing medium, Figure S9: Hierarchal cluster analysis of different MSNs based on physicochemical properties, intracellular fate, and penetration behavior, Figure S10. Heat map of the relationship between original physicochemical properties of MSN and the observable change after serum treatment, Table S1: The physicochemical properties of MSN, Table S2: Hydrodynamic size of MSNs measured under slightly acid culture medium, Table S3: Normalization values of all elements in the absence (top) and presence of serum (bottom) in the culture medium, Table S4: The list of Spearman correlation value between each group in the absence (top) and presence of serum (bottom) in the culture medium.

## References

[1] Wang Y., Sun S., Zhang Z., Shi D. (2018). Nanomaterials for Cancer Precision Medicine. Adv. Mater..

[2] Wilhelm S., Tavares A.J., Dai Q., Ohta S., Audet J., Dvorak H.F., Chan W.C.W. (2016). Analysis of nanoparticle delivery to tumours. Nat. Rev. Mater..

[3] Zan X., Hao P., Yang D., Feng S., Peng B., Appelhans D., Zhang T., Zan X. (2019). Designing nanoparticles with improved tumor penetration: Surface properties from the molecular architecture viewpoint. J. Mater. Chem. B.

[4] Lu H., Utama R.H., Kitiyotsawat U., Babiuch K., Jiang Y., Barner-Kowollik C. (2015). Enhanced transcellular penetration and drug delivery by crosslinked polymeric micelles into pancreatic multicellular tumor spheroids. Biomater. Sci..

[5] Sugahara K.N., Teesalu T., Karmali P.P., Kotamraju V.R., Agemy L., Greenwald D.R., Ruoslahti E. (2010). Coadministration of a Tumor-Penetrating Peptide Enhances the Efficacy of Cancer Drugs. Science.

[6] Cho M.H., Li Y., Lo P.-C., Lee H., Choi Y. (2020). Fucoidan-Based Theranostic Nanogel for Enhancing Imaging and Photodynamic Therapy of Cancer. Nano-Micro Lett..

[7] Lin Y.S., Tsai C.P., Huang H.Y., Kuo C.T., Hung Y., Huang D.M., Chen A.Y.C., Mou C.Y. (2005). Well-Ordered Mesoporous Silica Nanoparticles as Cell Markers. Chem. Mater..

[8] Chen Y.P., Chen H.A., Hung Y., Chien F.C., Chen P., Mou C.Y. (2012). Surface charge effect in intracellular localization of mesoporous silicananoparticles as probed by fluorescent ratiometric pH imaging. RSC Adv..

[9] Liu T.P., Wu S.H., Chen Y.P., Chou C.M., Chen C.T. (2015). Biosafety evaluations of well-dispersed mesoporous silica nanoparticles: Towards in vivo-relevant conditions. Nanoscale.

[10] Pratiwi F.W., Kuo C.W., Wu S.-H., Chen Y.-P., Mou C.Y., Chen P. (2018). The Bioimaging Applications of Mesoporous Silica Nanoparticles. Enzymes.

[11] Pratiwi F.W., Kuo C.W., Chen B.-C., Chen P. (2019). Recent advances in the use of fluorescent nanoparticles for bioimaging. Nanomedicine.

[12] Hosseinpour S., Walsh L.J., Xu C. (2020). Biomedical application of mesoporous silica nanoparticles as delivery systems: A biological safety perspective. J. Mater. Chem. B.

[13] Zhang L., Wang Y., Yang D., Huang W., Hao P., Feng S., Appelhans D., Zhang T., Zan X. (2019). Shape Effect of Nanoparticles on Tumor Penetration in Monolayers Versus Spheroids. Mol. Pharm..

[14] Matsuura R., Miyagawa S., Fukushima S., Goto T., Harada A., Shimozaki Y., Yamaki K., Sanami S., Kikuta J., Ishii M. (2018). Intravital imaging with two-photon microscopy reveals cellular dynamics in the ischeamia-reperfused rat heart. Sci. Rep..

[15] Tchoryk A., Taresco V., Argent R.H., Ashford M.B., Gellert P.R., Stolnik S., Grabowska A.M., Garnett M.C. (2019). Penetration and Uptake of Nanoparticles in 3D Tumor Spheroids. Bioconj. Chem..

[16] Takechi-Haraya Y., Goda Y., Sakai-Kato K. (2017). Control of Liposomal Penetration into Three-Dimensional Multicellular Tumor Spheroids by Modulating Liposomal Membrane Rigidity. Mol. Pharm..

[17] Huang K., Ma H., Liu J., Huo S., Kumar A., Wei T., Zhang X., Jin S., Gan Y., Wang P.C. (2012). Size-Dependent Localization and Penetration of Ultrasmall Gold Nanoparticles in Cancer Cells, Multicellular Spheroids, and Tumors in Vivo. ACS Nano.

[18] Hui Y., Yi X., Hou F., Wibowo D., Zhang F., Zhao D., Gao H., Zhao C.-X. (2019). Role of Nanoparticle Mechanical Properties in Cancer Drug Delivery. ACS Nano.

[19] Zhao Z., Ukidve A., Krishnan V., Mitragotri S. (2019). Effect of physicochemical and surface properties on in vivo fate of drug nanocarriers. Adv. Drug Deliv. Rev..

[20] Mapanao A.K., Voliani V. (2020). Three-dimensional tumor models: Promoting breakthroughs in nanotheranostics translational research. Appl. Mater. Today.

[21] Nunes A.S., Barros A.S., Costa E.C., Moreira A.F., Correia I.J. (2019). 3D tumor spheroids as in vitro models to mimic in vivo human solid tumors resistance to therapeutic drugs. Biotechnol. Bioeng..

[22] Ooft S.N., Weeber F., Dijkstra K.K., McLean C., Kaing S., Van Werkhoven E.D., Schipper L., Hoes L., Vis D.J., Van De Haar J. (2019). Patient-derived organoids can predict response to chemotherapy in metastatic colorectal cancer patients. Sci. Transl. Med..

[23] Muraca F., AlAhmari A., Giannone V.A., Adumeau L., Yan Y., McCafferty M.M., Dawson K.A. (2019). A Three-Dimensional Cell Culture Platform for Long Time-Scale Observations of Bio–Nano Interactions. ACS Nano.

[24] Li Y., Kumacheva E. (2018). Hydrogel microenvironments for cancer spheroid growth and drug screening. Sci. Adv..

[25] Denk W., Strickler J.H., Webb W.W. (1990). Two-photon laser scanning fluorescence microscopy. Science.

[26] Ban Z., Yuan P., Yu F., Peng T., Zhou Q., Hu X. (2020). Machine learning predicts the functional composition of the protein corona and the cellular recognition of nanoparticles. Proc. Natl. Acad. Sci. USA.

[27] Cai X., Dong J., Liu J., Zheng H., Kaweeteerawat C., Wang F., Ji Z., Li R. (2018). Multi-hierarchical profiling the structure-activity relationships of engineered nanomaterials at nano-bio interfaces. Nat. Commun..

[28] Silvestri A., Di Silvio D., Llarena I., Murray R.A., Marelli M., Lay L., Polito L., Moya S. (2017). Influence of surface coating on the intracellular behaviour of gold nanoparticles: A fluorescence correlation spectroscopy study. Nanoscale.

[29] Akoglu H. (2018). User’s guide to correlation coefficients. Turk. J. Emerg. Med..

[30] Nederman T., Norling B., Glimelius B., Carlsson J., Brunk U. (1984). Demonstration of an extracellular matrix in multicellular tumor spheroids. Cancer Res..

[31] Knight E., Przyborski S. (2015). Advances in 3D cell culture technologies enabling tissue-like structures to be createdin vitro. J. Anat..

[32] Sarkar S., Peng C.C., Kuo C.W., Chueh D.Y., Wu H.M., Liu Y.H., Chen P., Tung Y.C. (2018). Study of oxygen tension variation within live tumor spheroids using microfluidic devices and multi-photon laser scanning microscopy. RSC Adv..

[33] Wang H.X., Zuo Z.Q., Du J.Z., Wang Y., Sun R., Cao Z.T., Ye X.D., Wang J.L., Leong K.W., Wang J. (2016). Surface charge critically affects tumor penetration and therapeutic efficacy of cancer nanomedicines. Nano Today.

[34] Himmelstoß S.F., Hirsch T. (2019). Long-Term Colloidal and Chemical Stability in Aqueous Media of NaYF 4 -Type Upconversion Nanoparticles Modified by Ligand-Exchange. Part. Part. Syst. Charact..

[35] Chang R.L., Pratiwi F., Chen B.C., Chen P., Wu S.H., Mou C.Y. (2020). Simultaneous Single-particle Tracking and Dynamic pH Sensing Reveal Lysosome-targetable Mesoporous Silica Nanoparticles Pathways. ACS Appl. Mater. Interfaces.

[36] Sindhwani S., Syed A.M., Ngai J., Kingston B.R., Maiorino L., Rothschild J., Macmillan P., Zhang Y., Rajesh N.U., Hoang T. (2020). The entry of nanoparticles into solid tumours. Nat. Mater..

[37] Godet I., Shin Y.J., Ju J.A., Ye I.C., Wang G., Gilkes D.M. (2019). Fate-mapping post-hypoxic tumor cells reveals a ROS-resistant phenotype that promotes metastasis. Nat. Commun..

[38] Huang X., Zhuang J., Chung S.W., Huang B., Halpert G., Negron K., Sun X., Yang J., Oh Y., Hwang P.M. (2019). Hypoxia-tropic Protein Nanocages for Modulation of Tumor- and Chemotherapy-Associated Hypoxia. ACS Nano.

[39] Park C.C., Georgescu W., Polyzos A., Pham C., Ahmed K.M., Zhang H., Costes S.V. (2013). Rapid and automated multidimensional fluorescence microscopy profiling of 3D human breast cultures. Integr. Biol..

[40] Lynch I., Weiss C., Valsami-Jones E. (2014). A strategy for grouping of nanomaterials based on key physico-chemical descriptors as a basis for safer-by-design NMs. Nano Today.

[41] Xu M., Soliman M.G., Sun X., Pelaz B., Feliu N., Parak W.J., Liu S. (2018). How Entanglement of Different Physicochemical Properties Complicates the Prediction of in Vitro and in Vivo Interactions of Gold Nanoparticles. ACS Nano.

[42] Sujai P.T., Joseph M.M., Saranya G., Nair J.B., Murali V.P., Maiti K.K. (2020). Surface charge modulates the internalization vs. penetration of gold nanoparticles: Comprehensive scrutiny on monolayer cancer cells, multicellular spheroids and solid tumors by SERS modality. Nanoscale.

[43] Harush-Frenkel O., Rozentur E., Benita S., Altschuler Y. (2008). Surface Charge of Nanoparticles Determines Their Endocytic and Transcytotic Pathway in Polarized MDCK Cells. Biomacromolecules.

[44] Albanese A., Lam A.K., Sykes E.A., Rocheleau J.V., Chan W.C.W. (2013). Tumour-on-a-chip provides an optical window into nanoparticle tissue transport. Nat. Commun..

[45] Minchinton A.I., Tannock I.F. (2006). Drug penetration in solid tumours. Nat. Rev. Cancer.

[46] Chen D., Ganesh S., Wang W., Amiji M. (2020). Protein Corona-Enabled Systemic Delivery and Targeting of Nanoparticles. AAPS J..

[47] Chou C.C., Chen W., Hung Y., Mou C.Y. (2017). Molecular Elucidation of Biological Response to Mesoporous Silica Nanoparticles in Vitro and in Vivo. ACS Appl. Mater. Interfaces.

[48] Valente K.P., Suleman A., Brolo A.G. (2020). Exploring Diffusion and Cellular Uptake: Charged Gold Nanoparticles in an in Vitro Breast Cancer Model. ACS Appl. Bio Mater..

[49] Tsou C.J., Hsia C.H., Chu J.Y., Hung Y., Chen Y.P., Chien F.C., Chou K.C., Chen P., Mou C.Y. (2015). Local pH tracking in living cells. Nanoscale.

[50] Adair J.H., Suvaci E., Sindel J., Buschow K.H.J., Cahn R.W., Flemings M.C., Ilschner B., Kramer E.J., Mahajan S., Veyssière P. (2001). Surface and Colloid Chemistry. Encyclopedia of Materials: Science and Technology.

[51] Verwey E.J.W. (1947). Theory of the Stability of Lyophobic Colloids. J. Phys. Chem..

[52] Derjaguin B., Landau L. (1993). Theory of the stability of strongly charged lyophobic sols and of the adhesion of strongly charged particles in solutions of electrolytes. Prog. Surf. Sci..

[53] Mulvihill M.J., Habas S.E., Plante I.J.L., Wan J., Mokari T. (2010). Influence of Size, Shape, and Surface Coating on the Stability of Aqueous Suspensions of CdSe Nanoparticles. Chem. Mater..

[54] Aldana J., Lavelle N., Wang A.Y., Peng X. (2005). Size-Dependent Dissociation pH of Thiolate Ligands from Cadmium Chalcogenide Nanocrystals. J. Am. Chem. Soc..

[55] Oh J.Y., Kim H.S., Palanikumar L., Go E.M., Jana B., Park S.A., Kim H.Y., Kim K., Seo J.K., Kwak S.K. (2018). Cloaking nanoparticles with protein corona shield for targeted drug delivery. Nat. Commun..

[56] Huang K., Boerhan R., Liu C., Jiang G. (2017). Nanoparticles Penetrate into the Multicellular Spheroid-on-Chip: Effect of Surface Charge, Protein Corona, and Exterior Flow. Mol. Pharm..

[57] Lundqvist M., Stigler J., Elia G., Lynch I., Cedervall T., Dawson K. (2008). Nanoparticle size and surface properties determine the protein corona with possible implications for biological impacts. Proc. Natl. Acad. Sci. USA.

