# Supplementary material for: Evaluation of Nanoparticle Penetration in the Tumor Spheroid Using Two-Photon Microscopy
Source: Biomedicines. 2020 Dec 24;9(1):10. doi: 10.3390/biomedicines9010010 (PMC7824314; doi:10.3390/biomedicines9010010)
Supplement: Supplementary file 1 [file biomedicines-09-00010-s001.pdf]

Supporting Information

Article

Evaluation of Nanoparticle Penetration in the Tumor Spheroid Using Two-Photon Microscopy

Feby Wijaya Pratiwi [1], Chien-Chung Peng [1], Si-Han Wu [2], Chiung Wen Kuo [1], Chung-Yuan Mou [3], Yi-Chung Tung [1], Peilin Chen [1,*]

[1] Research Center for Applied Sciences, Academia Sinica, Taipei 11529, Taiwan
[2] Graduate Institute of Nanomedicine and Medical Engineering, Taipei Medical University, Taipei 11031, Taiwan
[3] Department of Chemistry, National Taiwan University, Taipei 10617, Taiwan
* Correspondence: peilin@gate.sinica.edu.tw

Imaging of extracellular matrix (ECM) surround the spheroid

After fixation of the spheroid in 4% PFA for one hour, the spheroids were then permeabilized with 0.25% Triton X-100 in PBS for 15 minutes and blocked with 3% BSA in PBS for another 1-h at room temperature. To demonstrate that our spheroids contain extracellular matrix (ECM), the spheroids were treated with antibody-related ECM- proteins marker, such as fibronectin, collagen IV, and vitronectin rabbit polyclonal antibody (Genetex, Taipei, Taiwan) at a concentration of 5μg/mL overnight at 4° C. After the washing steps, the spheroids were incubated with goat anti-rabbit secondary antibody conjugate Alexa-fluor ® 488 or Alexa-fluor ® 594 (1:1000 dilution) for 1-h at room temperature. The spheroid's images were obtained using a confocal microscope with the same setup described in the previous experiment.

Evaluation of MSN accumulation in the hypoxic region of the spheroid

The oxygen-sensitive dye (image-iT Red, H10498, Thermo Scientific Inc., Rochester, NY,USA) was employed to map the spheroid's oxygen variation. The oxygen-sensitive dye was added into the MG-63 cell suspension at a final concentration of 10 μM during the cells seeding time in the 96-well plate. After seven days for cell culture, the harvested spheroids were treated

with FITC labeled-MSN. A z-sectioned two-photon scanning was performed to evaluate MSN's ability (green) targeting the hypoxia region (red) in the spheroid at an excitation wavelength of 900 nm. The images obtained were then analyzed, and the colocalization percentage between these two fluorescences was calculated based on the Mander coefficient using Fiji Image J software.

Evaluation NP uptake in individual cells in spheroid

After 16 h of incubation with 0.5 mg/mL RITC-MSNs, the spheroids stained with Hoeschest were washed at least three times to remove the excess of NPs. The spheroids were dissociated by adding 50 uL 0.25% trypsin-EDTA and incubated for 15 min. Then, the solution was gently pipetted to separate individual cells. The live-cells were immediately imaged using a confocal microscope with a 10x objective at excitation wavelengths of 405 and 543 nm for Hoeschest and MSN, respectively. The percentages of cells contained MSN were obtained by calculating the numbers of cells containing MSNs(red color) over the total number of cells (blue).

Evaluation of MSN's intracellular fates

a. NP's entrapment in the lysosome

First, the 50 μg/mL of RITC-MSNs were incubated with 1μM of lysosome tracker (LysoTracker® Green DND-26, Thermo Scientific Inc., Rochester, NY) in the spheroid containing Hoeschest for 16 h to evaluate the NP entrapment in the lysosomes. After 16 h of incubation, the spheroids were washed at least three times to remove the excess of NPs and dyes. The living spheroid was then imaged using a confocal microscope with a 40x oil objective at excitation wavelengths of 405, 488, and 543 nm for Hoeschest, lysotracker, and MSN,

respectively. The images obtained were then analyzed, and the colocalization percentage between these two fluorescences was calculated based on the Mander coefficient using Fiji Image J software.

b. NP's in the recycling endosome

After 16 h of incubation with 50 μg/mL RITC-MSNs, the spheroids were washed at least three times to remove the excess of NPs and dyes and were fixed with 4% PFA for 1h. The spheroids were then permeabilized with 0.25% Triton X-100 in PBS for 15 minutes and blocked with 3% BSA in PBS for another 1-h at room temperature. The spheroids were then treated with Rab 11A rabbit polyclonal antibody (Invitrogen) at a concentration of 5μg/mL overnight at 4º C. After the washing steps; the spheroids were incubated with goat anti-rabbit secondary antibody conjugate Alexa-fluor ® 488 (1:1000 dilution) for 1-h at room temperature. The spheroid's images were obtained using a confocal microscope with the same setup described in the previous experiment.

Quantitative image analysis

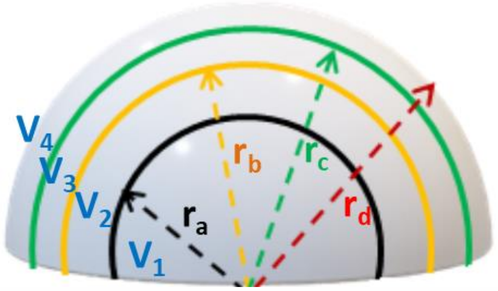

$$V_1 = V_2 = V_3 = V_4$$

$$\frac{4}{3}\pi {r_a}^3 = \frac{4}{3}\pi({r_b}^3 - {r_a}^3) = \frac{4}{3}\pi({r_c}^3 - {r_b}^3) = \frac{4}{3}\pi({r_d}^3 - {r_c}^3),$$

For example, diameter (D) of spheroid ~320 μm, therefore:

$$r_d = ½\ D;\ r_a = \frac{r_d}{\sqrt[3]{4}};\ \ r_b = r_a\sqrt[3]{2};\ c = r_a\sqrt[3]{3};$$

r_a~100 μm, r_b~127 μm, r_c~145 μm, r_d~160 μm

Region 1 is the volume in r_b, therefore, the thickness is about 100 µm (core region)

Region 2 is the rim volume in between r_a and r_b, therefore the thickness is about 27 µm

Region3 is the rim volume in between r_b and r_c, therefore, the thickness is about 18 µm

Region 4 is the rim volume in between r_c and r_d, therefore the, thickness is about 15 µm

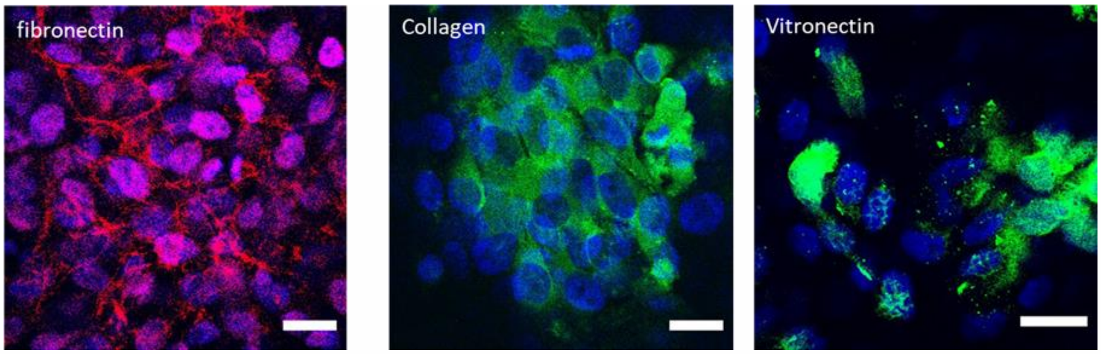

Figure S1. The immunostaining of cell spheroid with various protein marker commonly found in ECM such as fibronectin (red), collagen (green) and vitronectin (green). Scale bar 25 µm

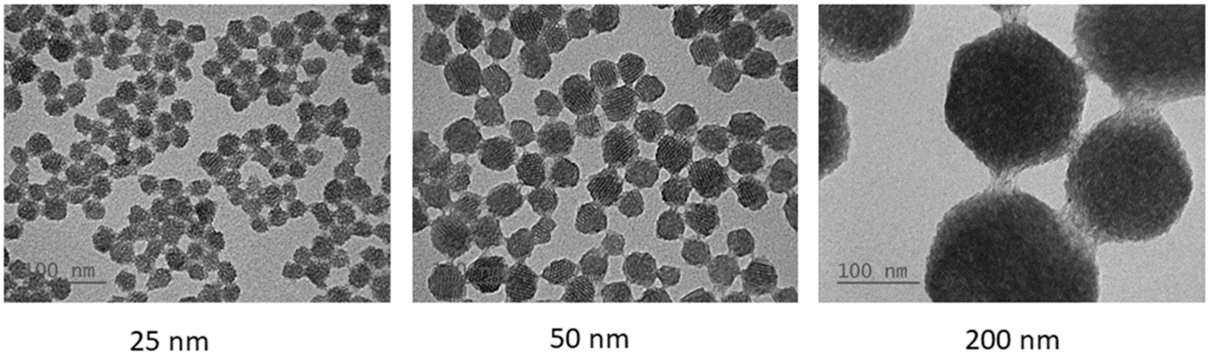

Figure S2. TEM micrograph of MSNs with different size 25, 50, and 200 nm. Scale bar=100 nm

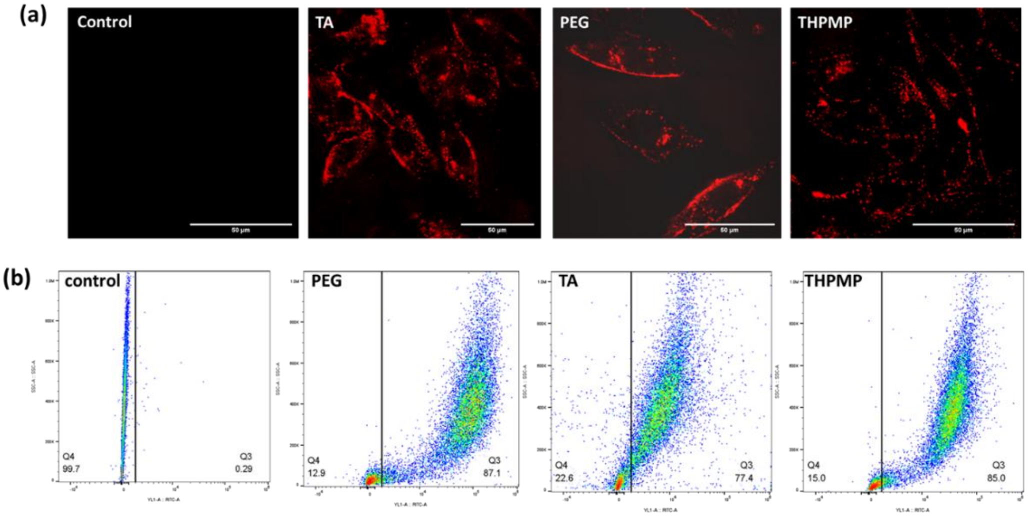

Figure S3. The confocal images (a) and flowcytometry (b) of different MSN uptake in monolayer cell culture

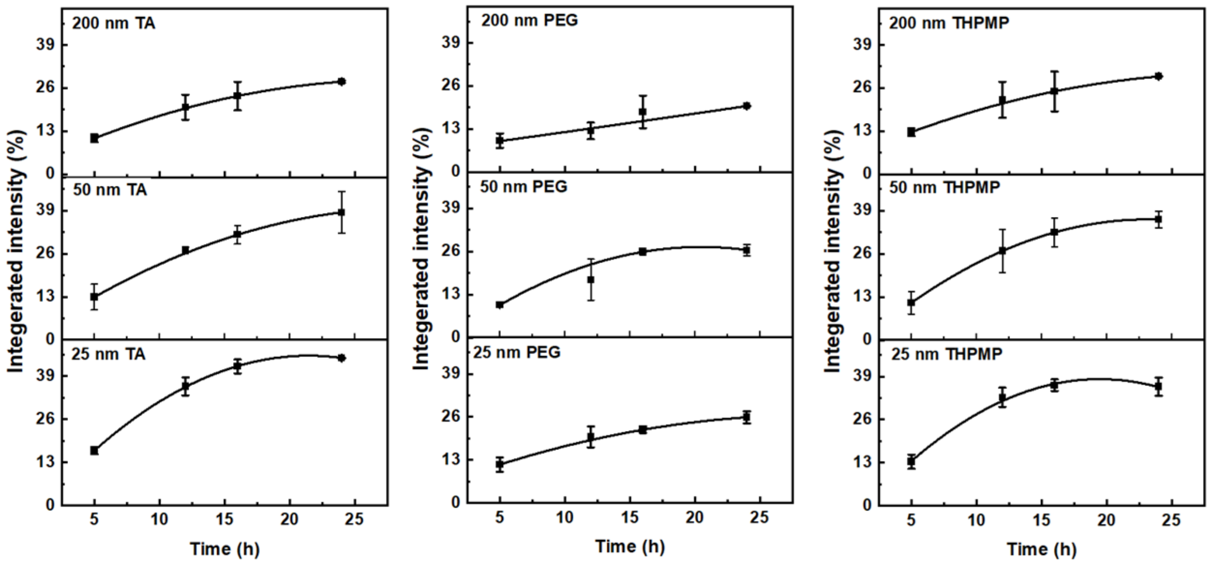

Figure S4. Time-dependent MSN's penetration into the core region of the spheroid. N=4-6

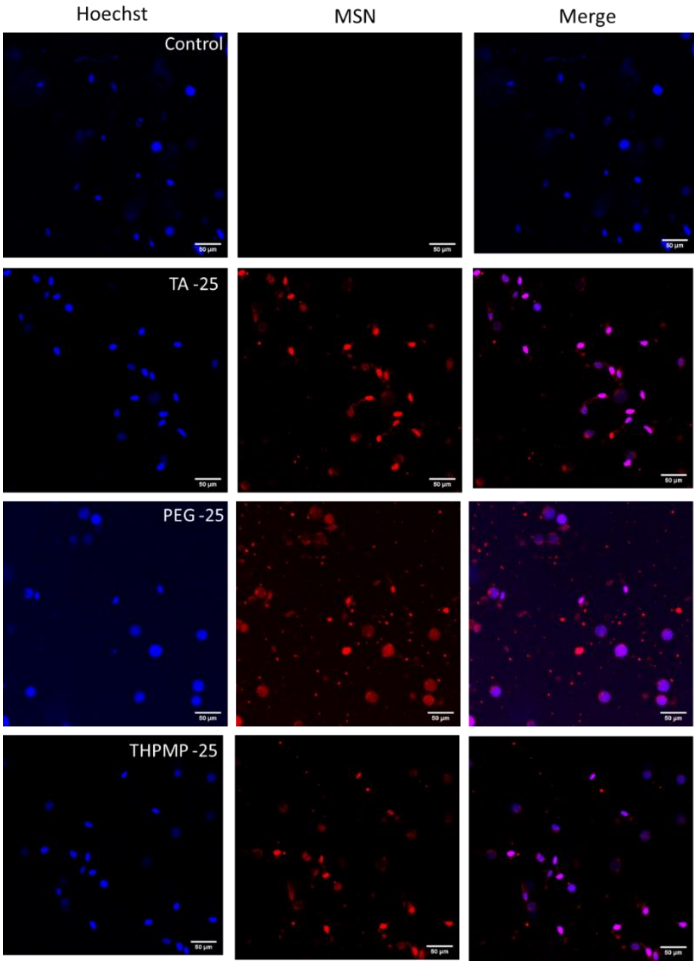

Figure S5. The confocal image of individual cells obtained from spheroids stained with Hoechst (blue) and nanoparticle (red)

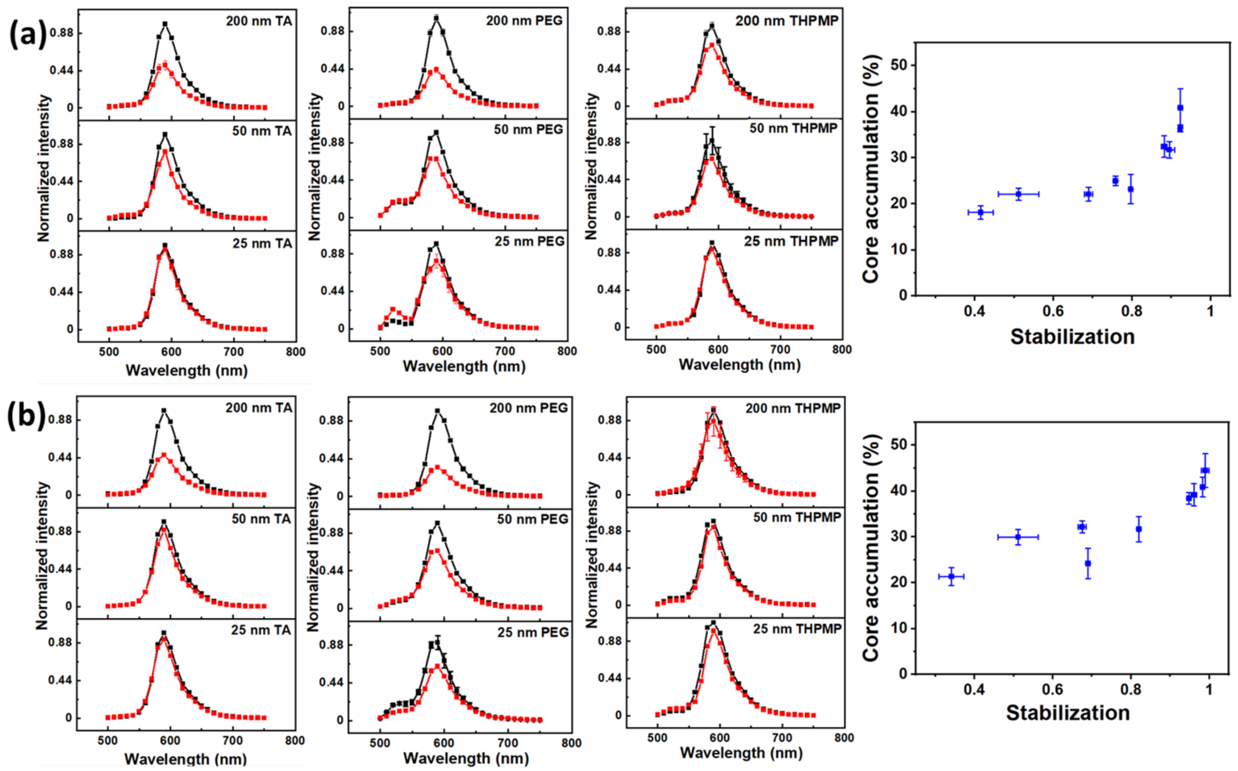

Figure S6. Stabilization of MSNs in slightly acid (a) free medium and (b) serum-containing medium at 0 (black line) and 16 h (red line). N=3

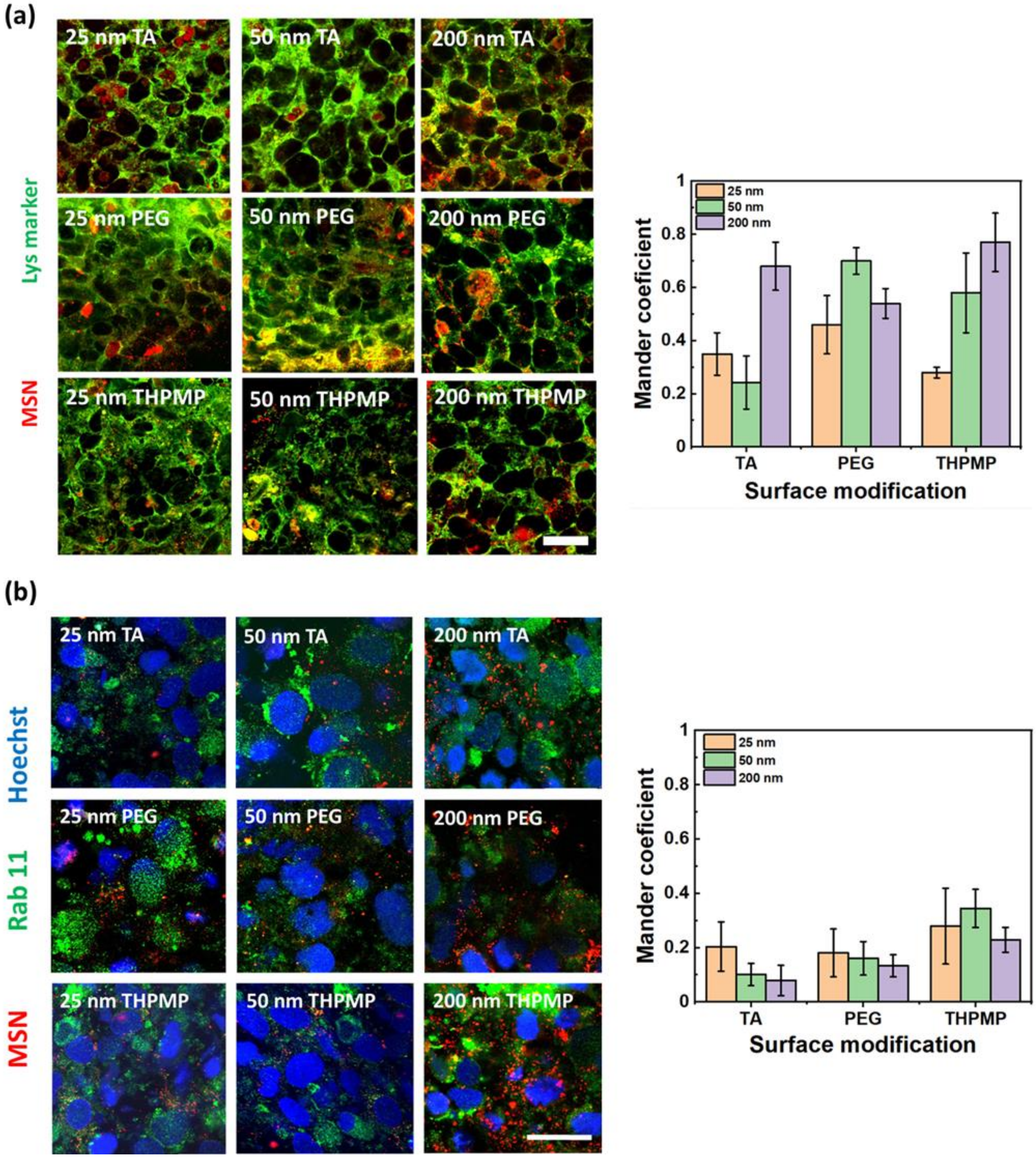

Figure S7. Colocalization of MSNs with (a) lysosome marker and (b) Rab11 (recycling endosome marker). Scale bar=25 μm N=3

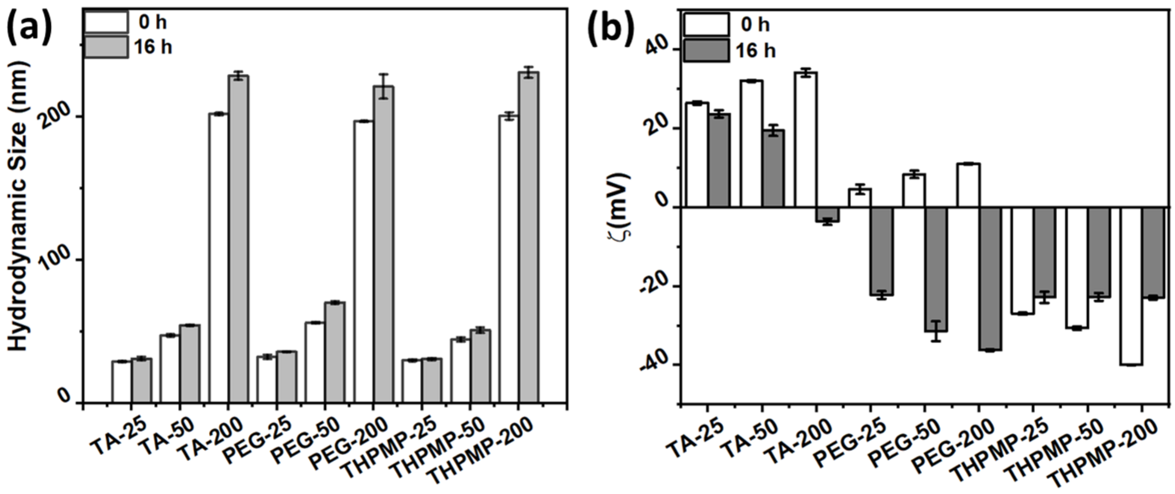

Figure S8. The MSN's (a) hydrodynamic size and (b) zeta potential change in the serum-containing medium. N=3

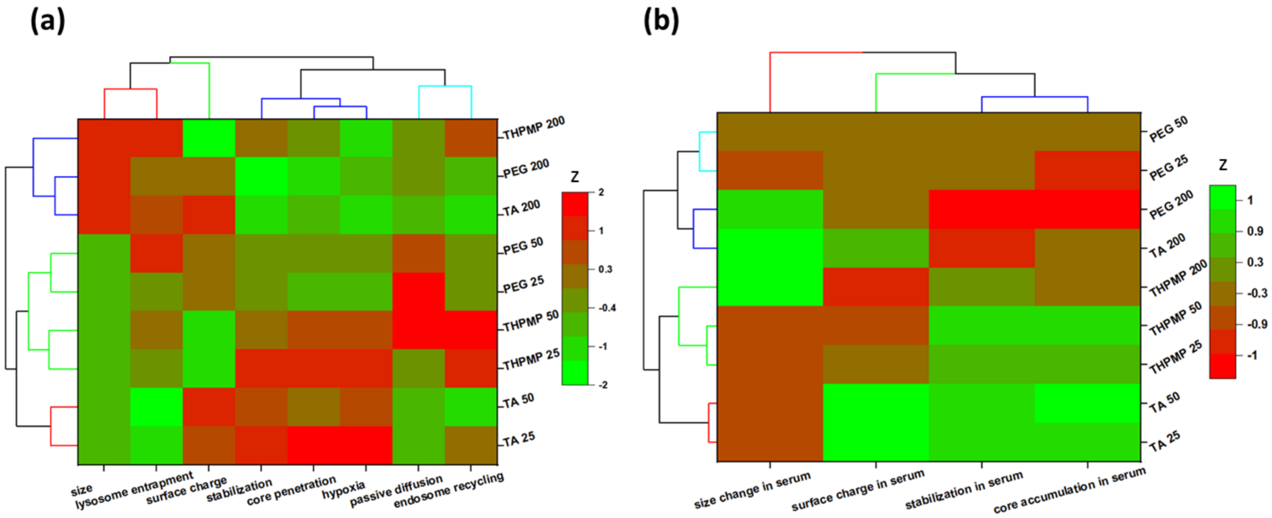

Figure S9. Hierarchal cluster analysis of different MSNs based on physicochemical properties, intracellular fate, and penetration behavior. The color map represents the normalized value of each element (z).

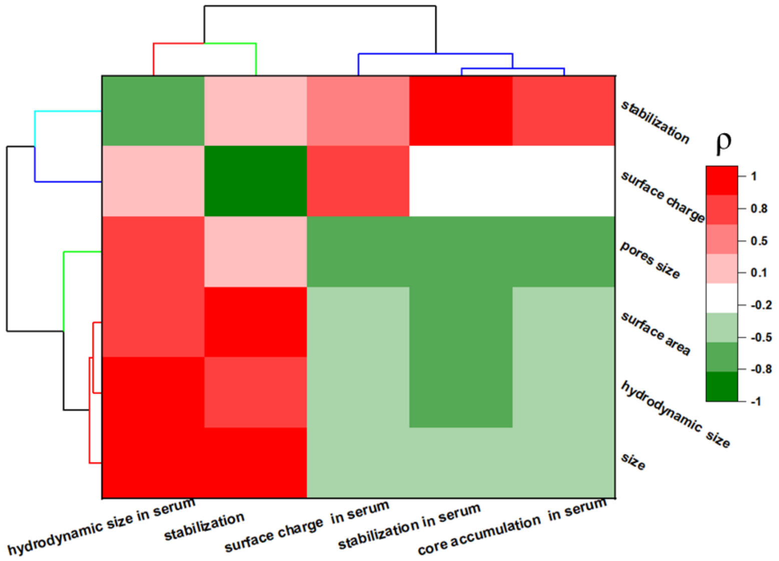

Figure S10. Heat map of the relationship between original physicochemical properties of MSN and the observable change after serum treatment. The color map represents the Spearmen coefficient value (ϱ).

Table S1. The physicochemical properties of MSN

Properties	PEG/TA			PEG			PEG/THPMP		
	25	50	200	25	50	200	25	50	200
[a]TEM size (nm)	22.5±2.9	49.1±4.7	201.6±10.3	21+3.4	47.7+4.9	186.7±15.7	23.6±7.3	41.5±9.1	192.7±28.5
[b]Hydro. Size (nm)	38.5±0.5	54.43±0.2	203±1.8	34.38±1.3	51.80±0.3	192.7±2.4	38.01±0.8	57.28±1.9	201.07±2.4
[c]Porous size (nm)	1.35	1.36	1.74	1.62	2.07	1.93	1.72	1.5	2.11
[d]Surf. area (m^2/g)	319.4	549.2	798.9	470.3	887.1	793.7	428.8	589.9	818.9
[e]Surf. charge (mV)	+26.03±0.7	32.1±0.6	34.23±0.5	+7.42±1.3	+9.12±0.6	+11.23±1.1	-26.67±0.6	-30.2±1.8	-39.9±0.8

[a] size was measured MSN's from TEM's micrograph. [b] Hydrodynamic sizes were measured based on the intensity distribution by dynamic light scattering (DLS) in DI water. [c]Pore sizes were determined using the Barrett−Joyner− Halenda (BJH) method. [d] surface charges were determined by zeta potential values in the DI water. [e] Surface area were calculated using the Brunauer−Emmett−Teller (BET) method.

Table S2. Hydrodynamic size of MSNs measured under slightly acid culture medium

MSN	hydrodynamic size (nm) / PDI	
	0 h	16 h
TA 25	29.34 / 0.11	31.77 / 0.18
TA 50	49.10 / 0.08	50.60 / 0.12
TA 200	201.93 / 0.05	208.89 / 0.13
THPMP 25	30.15 / 0.12	33.12 / 0.09
THPMP 50	49.68 / 0.16	53.56 / 0.25
THPMP 200	200.60 / 0.15	204.70 / 0.16
PEG 25	32.56 / 0.10	42.68 / 0.39
PEG 50	56.37 / 0.12	65.30 / 0.31
PEG 200	206.40 / 0.03	235.37 / 0.51

Table S3. Normalization values of all elements in the absence (top) and presence of serum (bottom) in the culture medium

	size	hydro size	surf. charge	stabilization	surf. area	core acc.	hypoxia pref.	passive diff.	endosomal rcyc.	lysosome entrap.
TA 25	-0.81	-0.76	0.84	1.15	-1.53	1.76	1.61	-0.77	0.16	-1.11
TA 50	-0.48	-0.55	1.05	0.87	-0.39	0.41	0.84	-0.77	-1.05	-1.74
TA 200	1.42	1.38	1.13	-1.05	0.84	-0.66	-1.12	-0.74	-1.30	0.84
THPMP 25	-0.82	-0.76	-1.04	1.01	-0.99	1.03	0.93	1.80	1.05	-0.11
THPMP 50	-0.49	-0.51	-1.17	0.19	-0.19	0.51	0.60	0.36	1.82	0.25
THPMP 200	1.31	1.36	-1.51	0.19	0.94	-0.47	-1.02	-0.51	0.45	1.37
PEG 25	-0.79	-0.81	0.17	-0.31	-0.78	-0.85	-0.55	-0.33	-0.11	-0.46
PEG 50	-0.57	-0.58	0.23	-0.16	1.28	-0.34	-0.48	1.50	-0.35	0.95
PEG 200	1.23	1.24	0.31	-1.90	0.82	-1.38	-0.80	-0.53	-0.67	0.01

	size	hydro. size	surf. charge	surf. area	pores size	stabilization	hydro.size	surf. charge	stabilization	core acc
TA 25	-0.81	-0.81	0.84	-1.53	-1.27	1.15	-0.81	1.71	0.92	0.86
TA 50	-0.48	-0.55	1.05	-0.39	-1.24	0.87	-0.56	1.51	0.95	1.29
TA 200	1.42	1.35	1.13	0.84	0.10	-1.05	1.34	0.44	-1.11	-0.47
THPMP 25	-0.82	-0.80	-1.04	-0.99	0.03	1.01	-0.82	-0.42	0.77	0.56
THPMP 50	-0.49	-0.56	-1.17	-0.19	-0.74	0.19	-0.60	-0.84	0.83	1.02
THPMP 200	1.31	1.33	-1.51	0.94	1.41	0.19	1.36	-1.06	0.22	-0.26
PEG 25	-0.79	-0.77	0.17	-0.78	-0.32	-0.31	-0.78	-0.44	-0.34	-1.29
PEG 50	-0.57	-0.47	0.23	1.28	1.27	-0.16	-0.39	-0.44	-0.40	-0.20
PEG 200	1.23	1.29	0.31	0.82	0.77	-1.90	1.26	-0.45	-1.84	-1.51

Grey indicates initial MSN's properties while black is post-serum incubation observable change

$$\text{Normalized Value} = \frac{[(\text{Value}) - \text{Mean (of all samples)}]}{\text{Standard deviation (of all samples)}}$$

Table S4. The list of Spearman correlation value between each group in the absence (top) and presence of serum (bottom) in the culture medium

	size	hydro size	surf. charge	absolute surf. charge	stabilization	surf. area	core acc.	hypoxia pref.	passive diff.	endosomal rcyc.	lysosome entrap.
**size**	1.00	0.93	0.22	0.60	-0.62	0.70	-0.60	-0.82	-0.40	-0.52	0.50
**hydro size**	0.93	1.00	0.15	0.70	-0.45	0.67	-0.50	-0.68	-0.33	-0.33	-0.42
**surf. charge**	0.22	0.15	1.00	-0.02	-0.15	-0.10	-0.10	0.00	-0.69	-0.85	-0.42
**absolute surf.charge**	0.60	0.70	-0.02	1.00	0.18	0.23	0.33	0.16	-0.31	0.00	-0.45
**stabilization**	-0.62	-0.45	-0.15	0.18	1.00	-0.62	0.95	0.87	-0.03	0.53	-0.45
**surf. area**	0.70	0.67	-0.10	0.23	-0.62	1.00	-0.52	-0.73	0.18	-0.30	0.85
**core acc.**	-0.60	-0.62	-0.10	0.33	0.95	-0.52	1.00	0.85	0.06	0.53	-0.32
**hypoxia pref.**	-0.82	-0.68	0.00	0.16	0.87	-0.73	0.85	1.00	0.03	0.42	-0.67
**passive diff.**	-0.40	-0.33	-0.69	-0.31	-0.03	0.18	0.06	0.03	1.00	0.57	0.43
**endosomal rcyc.**	-0.52	-0.33	-0.85	0.00	0.53	-0.30	0.53	0.42	0.57	1.00	0.12
**lysosome entrap.**	0.50	0.58	-0.42	0.28	-0.45	0.85	-0.32	-0.67	0.43	0.12	1.00

	size	hydro. size	surf. charge	surf. area	pores size	stabilization	hydro.size	surf. charge	stabilization	core acc.
**size**	**1.00**	**0.93**	**0.22**	**0.70**	**0.47**	**-0.62**	**0.93**	**-0.32**	**-0.42**	**-0.32**
**hydro. size**	**0.93**	**1.00**	**0.15**	**0.87**	**0.72**	**-0.72**	**0.97**	**-0.37**	**-0.62**	**-0.45**
**surf. charge**	**0.22**	**0.15**	**1.00**	**-0.10**	**-0.33**	**-0.15**	**0.08**	**0.78**	**-0.08**	**0.00**
**surf. area**	**0.70**	**0.87**	**-0.10**	**1.00**	**0.82**	**-0.62**	**0.87**	**-0.50**	**-0.58**	**-0.33**
**pores size**	**0.47**	**0.72**	**-0.33**	**0.82**	**1.00**	**-0.60**	**0.70**	**-0.60**	**-0.73**	**-0.62**
**stabilization**	**-0.62**	**-0.72**	**-0.15**	**-0.62**	**-0.60**	**1.00**	**-0.65**	**0.43**	**0.90**	**0.83**
**hydro.size**	**0.93**	**0.97**	**0.08**	**0.87**	**0.70**	**-0.65**	**1.00**	**-0.42**	**-0.53**	**-0.42**
**surf. charge**	**-0.32**	**-0.37**	**0.78**	**-0.50**	**-0.60**	**0.43**	**-0.42**	**1.00**	**0.37**	**0.38**
**stabilization**	**-0.42**	**-0.62**	**-0.08**	**-0.58**	**-0.73**	**0.90**	**-0.53**	**0.37**	**1.00**	**0.90**
**core acc**	**-0.32**	**-0.45**	**0.00**	**-0.33**	**-0.62**	**0.83**	**-0.42**	**0.38**	**0.90**	**1.00**

Highlight yellow, orange, and green indicate fair, moderate, and strong correlation between the groups, respectively.
